# Changes in Plasma Levels of ADAMTS13 and von Willebrand Factor in Patients Undergoing Elective Joint Arthroplasty

**DOI:** 10.3390/jcm11216436

**Published:** 2022-10-30

**Authors:** Jun Kit He, Samuel Schick, Marshall Williams, Bradley Wills, Martim Pinto, Gean Viner, Eugene Brabston, Amit Momaya, X. Long Zheng, Brent Ponce

**Affiliations:** 1 Department of Orthopaedic Surgery, The University of Alabama at Birmingham, Birmingham, AL 35249, USA; 2 Hughston Clinic, Columbus, GA 31909, USA; 3 Department of Pathology and Laboratory Medicine, University of Kansas Medical Center, Kansas City, KS 66160, USA; 4 Institute of Reproductive Medicine and Developmental Sciences, University of Kansas Medical Center, Kansas City, KS 66160, USA

**Keywords:** ADAMTS13, von Willebrand factor, surgery, arthroplasty

## Abstract

**Background:** The risk of venous thromboembolic events (VTE) increases in patients undergoing total shoulder arthroplasty (TSA). However, there is no guidelines for prophylaxis. A decreased ratio of ADAMTS13 to VWF has been reported in patients with VTE. This study evaluates how TSA affects this ratio to better characterize timing of VTE risk and develop better guidelines for prophylactic treatment. **Methods:** Patients receiving TSA between 2016 and 2019 were recruited for this study following informed consent. Blood samples were collected at the clinic visit prior to surgery, postoperatively within one hour, at 24 h, 48 h, 2 and 6 weeks. Plasma levels of ADAMTS13 activity and VWF antigen were determined with a FRETS-VWF73 and an enzyme-linked immunoassay, respectively. **Results:** Of 22 patients included in the study, the mean age (± SD) was 68 ± 11 years. The most common diagnosis and surgery were osteoarthritis (68%) and reverse TSA (77%), respectively. Plasma ADAMTS13 activity was reduced immediately following surgery and remained lower than the baseline until postoperative day 2 (POD-2) (93.7 ± 28.5 IU/dL, *p* = 0.009). VWF antigen was the highest on POD-2 (253.2 ± 101.0%, *p* = 0.0034). The ADAMTS13/VWF ratio followed the same pattern, lowest on POD-2 (0.41 ± 0.20, *p* = 0.0016). All levels returned to baseline by two weeks. **Conclusions:** TSA resulted in low ADAMTS13 activity and high VWF acutely post-surgery day 2, suggesting that risk for VTE may be the highest during this period. ADAMTS13/VWF ratio is a useful marker to identify patients who may need proper anticoagulation after TSA.

## 1. Introduction

Venous thromboembolism (VTE), including deep vein thrombosis (DVT) and pulmonary embolism (PE), is a dire complication of surgery. Symptomatic VTE can lead to mortality or long-term morbidity such as pulmonary hypertension, recurrence of thrombosis, and post-thrombotic syndrome in addition to readmission or delayed discharge [1,2]. Patients can present after discharge with fatal PE as the first clinical manifestation. Reliance on early diagnosis and treatment is imperative [3]. Cost of treatment for VTE in the United States can reach $33,000 per case showing the high burden of this pathology [4]. The advent of prophylactic measures has drastically decreased the rate of clinically significant VTE, but there is a small subset of patients who still develop VTE despite these efforts. Further understanding of the pathology behind VTE is needed for development of effective and focused regimens [5,6].

Total shoulder arthroplasty (TSA) has increased 3.7-fold from 2000 to 2010 for glenohumeral osteoarthritis and is the third most common joint replacement after hip and knee [1,7]. Patients undergoing orthopedic surgery are at increased at risk of VTE due to vessel trauma, venous stasis from immobility, and a relatively elderly population [8,9]. After TSA, symptomatic VTE rates reported in the literature range from 0.24% to 2.6% [10,11,12,13,14,15,16,17,18,19]. VTE prophylaxis after upper extremity is not well-established because of the low incidence and insufficiency of quality studies that address this issue [13,20].

Multivariate logistic regression analysis of 83 of 13,299 patients with VTE revealed that hypoalbuminemia (albumin level < 3.5 g/dL), an increased length of stay, and African American ethnicity were independent risk factors for VTE development [21]. However, no specific coagulation factor was identified to be associated with VTE following TSA. Understanding the mechanism and timing of post-surgical coagulability is necessary for prophylaxis decision-making given the lack of standardized guidelines.

von Willebrand factor (VWF), a large adhesion glycoprotein, is synthesized in vascular endothelial cells and megakaryocytes [22]. It may be constitutively secreted and acutely released upon stimulation into blood where it acts as a carrier protein that protects clotting factor VIII from degradation by activated protein C [23,24,25]. Endothelial VWF plays an important role for normal hemostasis and its elevation has been associated with arterial [26,27,28] and venous thromboembolism [29]. The function of VWF is tightly regulated by a plasma metalloprotease, ADAMTS13 (A Disintegrin And Metalloproteinase with ThromboSpondin type 1 repeats, member, 13), first identified and cloned in 2001 [30,31]. ADAMTS13 cleaves VWF at the Tyr1605-Met1606 bond, thus inhibiting platelet agglutination and thrombus formation [32,33,34].

Severe deficiency plasma ADAMTS13 activity may lead to increased size of VWF multimers and increased risk of microvascular thrombosis such as thrombotic thrombocytopenic purpura [35,36,37]. The ratio of ADAMTS13 to VWF is also a vital indicator of the functionality of the coagulation system. Changes from baseline have been described with operations such as aortic surgery, coronary artery bypass grafting, hepatectomy, and liver transplantation, and have been suggested to play a crucial role in complications including organ dysfunction and thrombosis [38,39,40,41,42]. Specifically, in orthopedic surgery, in a series of 93 patients undergoing total hip (THA) or knee arthroplasty (TKA), VWF antigen and activity were acutely increased while ADAMTS13 and its activity were decreased from preoperative levels [20]. In some cases, an intensive hemostatic treatment may increases the risk of thrombosis in patients with coagulopathy [43].

A better understanding of the physiologic changes affecting coagulation is needed to direct guidelines for VTE prophylaxis in shoulder replacement surgery [44]. This study evaluates the changes in ADAMTS13 and VWF in patients undergoing TSA with both preoperative and five acute postoperative timepoints. Our results demonstrate that there was an acute increase in plasma VWF and a decrease in plasma ADAMTS13 levels, but both return to normal by six weeks post-operatively.

## 2. Materials and Methods

Institutional Review Board approval was obtained from the University of Alabama at Birmingham for this prospective observational cohort study.

### 2.1. Participants

Consenting patients scheduled to receive a total shoulder arthroplasty from a single surgeon between 2016 and 2019 were recruited for this study. Inclusion criteria included age of 18 years or older, indication for total shoulder replacement, and healthy enough to undergo surgery. Patients were excluded if they could not undergo surgery, were having revision surgery, were pregnant, had active infection, or had an active known clotting disorder or another hematologic pathology.

### 2.2. Sample Collection and Measurement

Blood samples were collected at six perioperative time points: at the clinic visit prior to surgery, postoperatively within one hour, 24 h, 48 h, two weeks, and six weeks. Samples were transported to the laboratory in sodium citrated tubes (nine parts venous blood, one part 0.106 mol/L sodium citrate). The plasma was extracted after centrifugation at 3500 xg for 15 min and the sample was stored at −80 °C.

### 2.3. Assay for Plasma ADAMTS13 Activity

A recombinant FRETS-VWF73 assay was used to assess plasma ADAMTS13 activity as described previously [45,46,47]. The activity was determined against a standard reference curve. The reference curve was created using pooled normal human plasma in serial dilutions. Normal ADAMTS13 activity was defined as 100 IU/dL.

### 2.4. Assay for Plasma VWF Antigen

An enzyme-linked immunosorbent assay (ELISA) was used to measure plasma VWF antigen levels as previously described [47,48]. Nine-six-well plates were coated with either rabbit polyclonal anti-human VWF antibodies (Dako, Denmark) at 4 °C overnight. Phosphate buffered saline (PBS) containing 2.5% bovine serum albumin (BSA) and 0.05% Tween 20 was used to block the non-specific binding sites on the plates for 30 min. The blocking buffer was decanted. After adding the diluted plasma samples to the plates, they were incubated for two hours at 37 °C. Next, there was decanting of the samples and washing with PBS three times. Rabbit polyclonal anti-human VWF conjugated with horseradish peroxidase (Dako) was diluted to 1:3000 with phosphate-buffered saline with 0.1% tween-20 (PBST) and 0.5% BSA. This was added to the wells and incubated at 37 °C for one hour. Washing was repeated. Tetramethylbenzidine (TMB) was added to the wells. The samples were observed for a color change reaction and stopped after 5 to 10 min with 0.5 M H_2_SO_4_. A spectrophotometer was used to measure optical density at 450 nm. A standard reference curve was used to assess the samples. This was done using pooled normal human plasma in serial dilutions. A normal VWF antigen level was defined as 100%.

### 2.5. Clinical Data Collection

Clinical data was retrospectively collected through chart review. This included patient demographics and past medical history. History of clotting or conditions that would put a participant at risk for future clots were specifically noted.

### 2.6. Statistical Analysis

Medians (95% confidence interval) were determined using Prism7 (GraphPad Software, La Jolla, California, United States). A Kruskal–Wallis test or Mann–Whitney test was used to assess differences between ≥3 groups or 2 groups. A *p*-value of less than 0.05 and 0.001 was considered significant and highly significant, respectively. 

## 3. Results

### 3.1. Patient Population

During the study period, a total of 22 patients were enrolled into the study. Mean age (±SD) was 68 ± 11 years. The study included 15 males and 7 females. There were a similar number of right- (n = 10) and left- (n = 12) sided operations. The most common pathologies were osteoarthritis (15/22, 68%) and rotator cuff tear with or without arthropathy (12/22, 55%). Most surgeries were reverse total shoulder arthroplasties (17/22, 77%) (Table 1). No patients developed DVT during the study period.

### 3.2. Plasma Levels of ADAMTS13 Activity, VWF Antigen and Ratio of ADAMTS13/VWF

**Figure 1:**
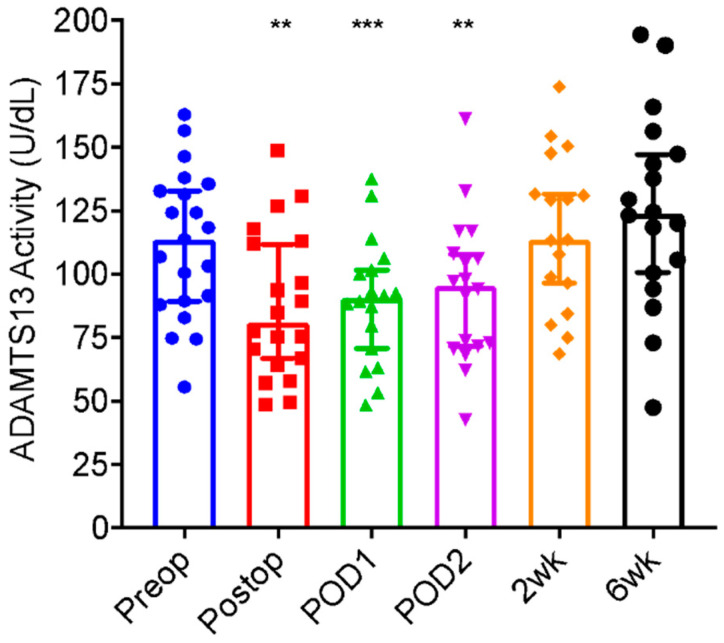
ADAMTS13 (A Disintegrin And Metalloprotease with Thrombospondin type 1 repeats, 13) activity in patients before and after Joint Arthroplasty. Plasma levels of ADAMTS13 activity in patients before (Preop), immediately (postop), one day (POD1), two days (POD2), two weeks (2 wk), and six weeks (6 wk) after elective joint arthroplasty. Each dot represents a result from each patient. The vertical bars are the medians (±95% confidence interval). Here, **, and *** indicate *p* < 0.01 and 0.001, respectively.

Median plasma ADAMTS13 activity was 113.7 IU/dL preoperatively. There was a significant drop in plasma levels of ADAMTS13 activity immediately (87.7 ± 28.9 IU/dL, *p* = 0.0021), one day (89.4 ± 24.4 IU/dL, *p* = 0.0003), and two days (93.7 ± 28.5 IU/dL, *p* = 0.009) after operation. Each subsequent postoperative day, plasma ADAMTS13 activity levels were increasing towards normal. At two weeks (116.7 ± 30.4 IU/dL, *p* = 0.567) and six weeks (125.4 ± 38.2, *p* = 0.266), the plasma ADAMTS13 levels were not significantly different from baseline (Table 2 and Figure 1).

**Figure 2:**
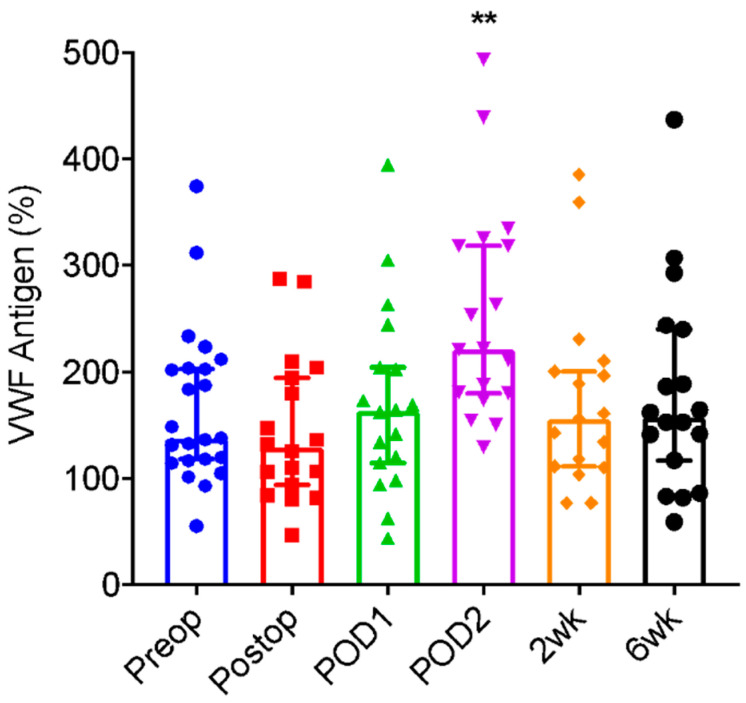
von Willebrand factor (VWF) antigen in patients before and after Joint Arthroplasty. Plasma levels of VWF antigen in patients before (Preop), immediately (postop), one day (POD1), two days (POD2), two weeks (2 wk), and six weeks (6 wk) after elective joint arthroplasty. Each dot represents a result from each patient. The vertical bars are the medians (±95% confidence interval). Here, ** indicates *p* < 0.01.

Median baseline plasma VWF antigen was 138.1%. Compared to mean (±SD) preoperative VWF antigen 167.5 (±73.5)%, antigen level was significantly higher than baseline at two days following operation 253.2 (±101.0)% (*p* = 0.0034). Immediately after operation 145.1 (±68.7)% (*p* = 0.248) and one day after operation 172.1 (±87.9)% (*p* = 0.632) antigen levels were not significantly different from the pre-operative state. By two weeks 174.5 (±87.6)% (*p* = 0.425) and six weeks 180.1 (±95.2%) (*p* = 0.487) after operation, levels returned to baseline (Table 2 and Figure 2).

**Figure 3:**
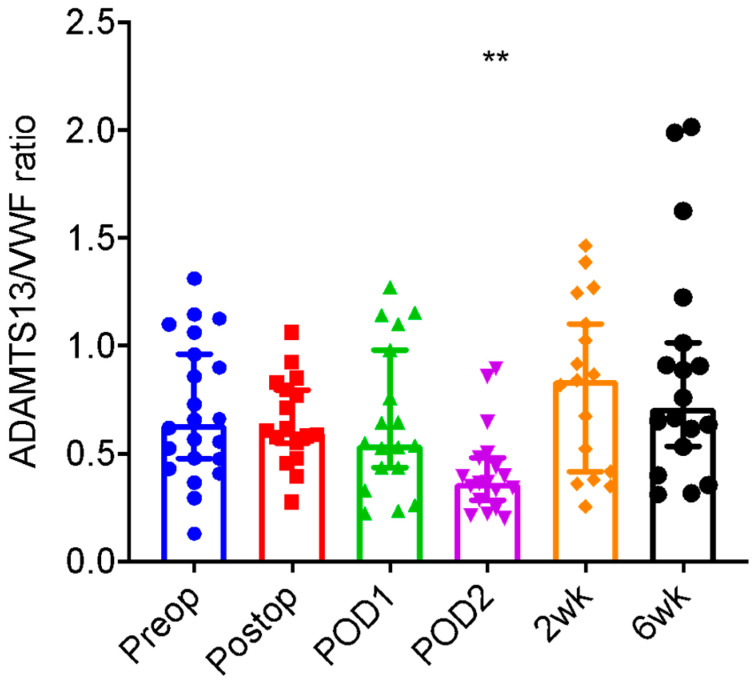
A Disintegrin and Metalloprotease with Thrombospondin type 1 repeats, 13/von Willebrand factor (ADAMTS13/VWF) ratios in patients before and after joint arthroplasty. Plasma ADAMTS13/VWF ratios in patients before (Preop), immediately (postop), one day (POD1), two days (POD2), two weeks (2 wk), and six weeks (6 wk) after elective joint arthroplasty. Each dot represents a result from each patient. The vertical bars are the medians (±95% confidence interval). Here, ** indicates *p* < 0.01.

An analogous pattern was seen with the ADAMTS13/VWF ratio. A significantly lower median ratio (± SD) (resulting from lower ADAMTS13 activity and higher VWF antigen) from the preoperative ratio of 0.70 (± 0.32) was seen on the second postoperative day (0.41 ± 0.20) (*p* = 0.0016). The immediate postoperative (0.65 ± 0.34) (*p* = 0.338) and one day postoperative (0.65 ±0.34) (*p* = 0.120) ratios were not a significant change from baseline, although trending downward. At two weeks (0.82 ±0.39) (*p* = 0.647) and six weeks (0.88 ±0.53) (*p* = 0.976), the ratios returned to baseline (Table 2 and Figure 3). These results suggest the possible immediate consumption of VWF during surgical procedure and acute phase reaction that drives VWF synthesis and secretion subsequently within the next 48 h following surgery.

## 4. Discussion

VTE is a known complication of surgery that remains as a notable cause of morbidity and mortality. Several studies have found that changes in the ratio of VWF to ADAMTS13 impact thrombosis potential in patients with various diseases [49,50,51,52,53]. The results of this study are consistent with the changes described in the literature and the relationship between VWF, ADAMTS13, and other surgeries [20,29,54,55,56,57,58]. Plasma VWF antigen levels were significantly higher than preoperative levels acutely while plasma ADAMTS13 activity was decreased significantly. This results in a significantly reduced ratio of ADAMTS13 to VWF immediately following surgery and on POD2, further demonstrating the imbalance from surgical exacerbation. By two weeks after surgery, plasma VWF and ADAMTS13, as well as the ratio between the two all returned to baseline. This supports that the hemodynamic changes predisposing patients to thrombosis are acute and suggests that early chemical prophylaxis such as anti-VWF (caplacizumab) or recombinant ADAMTS13 (once it becomes available) may be beneficial in preventing VTE after TSA.

VWF and ADAMTS13 are important factors in maintaining the balance between hemostasis and thrombosis [34,59,60]. VWF promotes platelet adhesion and aggregation while ADAMTS13 cleaves VWF multimers to prevent excessive formation of VWF-platelet aggregates and thrombosis. Deviation from normal levels and activity in each, in the form of increased VWF or decreased ADAMTS13 activity, may be associated with an increased risk of thrombosis, exemplified by thrombotic thrombocytopenic purpura (TTP) [61,62] and thrombosis associated with cancer [63], systemic lupus erythematosus, and systemic sclerosis [64,65,66]. Reiter et al. (2003) demonstrated an inverse relationship between VWF and ADAMTS13 levels after inducing release of VWF multimers with desmopressin and inflammation [67,68]. Karakaya et al. (2016) conducted a case–control study in which participants with and without VTE were age- and gender-matched and those with medical conditions affecting VWF and ADAMTS13 levels were excluded. The VTE group had significantly higher VWF levels and lower ADAMTS13 levels [55]. In contrast, Mazetto et al. (2012) found in their case–control study that both VWF and ADAMTS13 levels were elevated in VTE patients as well as TNFa and IL-6. They suggested that ADAMTS levels could be compensatory against persistently elevated VWF levels and that inflammation may also be a risk for VTE [29].

Liu et al. (2015) studied the changes of procoagulant and fibrinolytic factors in 51 total hip arthroplasty (THA) patients and 42 total knee arthroplasty (TKA) patients. They found that VWF antigen and activity were increased during POD1 and POD2 while plasma ADAMTS13 antigen and activity were decreased continuously from preoperative levels until POD7. Antithrombin III was also decreased while fibrinogen and D-dimer were increased in the week following surgery but no changes were shown in prothrombin time (PT), activated partial thromboplastin time (APTT), and thrombin time (TT) [20]. Our data expands on this and demonstrates that ADAMTS13 levels return to normal around 2 weeks after surgery. The timing of diagnosis for VTE after total hip and total knee arthroplasty cases are reported to be between 17–34 days and 7–20 days postoperatively, respectively [69,70]. Although different joints than the shoulder, this reflects the acute postoperative nature of VTE, consistent with our results and encapsulated by our 6-week follow-up.

Russell et al. (2018) described VWF and ADAMTS13 levels in 106 pediatric trauma patients. They found a similar higher median plasma level of VWF and low ADAMTS13 activity/VWF antigen ratio after trauma [47]. This ratio was also significantly lower in patients with higher injury severity scores (ISS). However, they also reported that there was no direct correlation between endothelial damage and ADAMTS13 activity, suggesting that the inhibition of ADAMTS13 is multifactorial in cause [47].

Symptomatic VTE rates after TSA range from 0.24% to 2.6% [10,11,12,13,14,15,17,18,19,21,71]. The pathogenesis of VTE involves the disturbances of Virchow’s triad: vessel wall injury, blood hypercoagulability, and venous stasis [1]. This coagulation activation and dysfunction may be caused by surgery and anesthetics [20]. With shoulder arthroplasty, traction on the arm has been suggested to cause endothelial injury that leads to thrombus formation. In particular, the axillary vein sustains damage with repeated rotation of the humerus and traction and pressure from retractors during glenoid exposure during the surgery. Furthermore, intramedullary reaming can cause embolic shower [72]. The beach chair position also leads to venous stasis as the lower extremities are dependent. Finally, operation for trauma and elderly population with medical comorbidities such as history of VTE, obesity, estrogen use, and congestive heart failure also increase the risk of VTE [73].

Regarding prophylaxis, current guidelines recommend an individualized approach, balancing probability of VTE with bleeding risk [22]. The 2009 American Academy of Orthopaedic Surgeons (AAOS) recommendation was for, in general, use of mechanical and/or chemoprevention in shoulder arthroplasty patients. Mechanical prophylaxis is safe and should be considered in all patients while chemoprevention should be weighed with the patient’s risks. In terms of type of chemoprophylaxis, the National Institute for Health and Care Excellence (NICE) in 2010 suggests the use of heparin or low-molecular-weight heparin until patient mobility is restored [11].

As a preliminary case series, this study was limited by a low number of patients with a heterogeneous set of medical comorbidities. Factors such as demographics, type of shoulder arthroplasty, and medical history could not be evaluated regarding the role they play in affecting VWF and ADAMTS13 levels in this population. None of the participants in the study developed symptomatic VTE. Although the effects imposed by surgery were described, the levels of VWF and ADAMTS13 may change more dramatically or in an unexpected manner in overt thrombosis. Future research will focus on the effect of patient characteristics and medications as well as compare levels in patients who develop VTE to those in patients who do not.

## 5. Conclusions

Decreased ADAMTS13 activity and increase in VWF antigen levels place patients at increased risk for VTE complications. The purpose of this study was to assess the changes of these in patients undergoing shoulder arthroplasty. The ratio of ADAMTS13 to VWF declined until it was significantly lower than baseline at two days post-operatively, suggesting that shoulder surgery escalates the coagulable state acutely but returns to normal by two weeks. This suggests that early chemical prophylaxis may be beneficial in helping prevent VTE in patients receiving TSA. While further studies are necessary to clarify the precise time period, population, and type of VTE prophylaxis, ADAMTS13 and VWF levels are crucial to describing the physiologic changes that take place and can be useful in identifying those at greatest risk of VTE.

## Figures and Tables

**Table 1:** Patient characteristics.

Characteristic	N = 22
Age (years)	
Mean ± SD	68 ± 11
Range	41–84
Gender	15/7
Male/Female	
Operative Shoulder (R/L)	10/12
Procedure	
Anatomic Total Shoulder Arthroplasty	3
Reverse Total Shoulder Arthroplasty	17
Pre-operative sample only	2

SD, standard deviation; R/L, right/left.

**Table 2:** Plasma levels of ADAMTS13 (A Disintegrin And Metalloprotease with Thrombospondin type 1 repeats, 13), von Willebrand factor (VWF), and ADAMTS13/VWF ratios in patients before and after joint arthroplasty.

**ADAMTS13 Activity (IU/dL)**						
	**Pre-Operative**	**Immediate Post-Operative**	**POD 1**	**POD 2**	**2 Weeks**	**6 Weeks**
N	21	20	18	18	17	18
Median	113.7	81.0	90.4	95.5	113.5	123.8
Range	55.4–162.7	48.3–148.5	48.5–137.6	42.5–161.1	68.5–173.6	47.4–194.3
Mean ± SD	111.9 ± 28.9	87.7 ± 28.9	89.4 ± 24.4	93.7 ± 28.5	116.7 ± 30.4	125.4 ± 38.2
*p*-Value (Δ from Preoperative value)	-	**0.0021**	**0.0003**	**0.0010**	0.567	0.266
VWF Antigen (%)						
	Pre-Operative	Immediate Post-Operative	POD 1	POD 2	2 weeks	6 Weeks
N	23	18	18	18	17	18
Median	138.1	128.9	163.8	221.4	156.2	157.6
Range	55.6–374.9	46.8–287.7	44.0–394.7	129.3–493.4	77.5–385.7	59.6–437.1
Mean ± SD	167.5 ± 73.5	145.1 ± 68.7	172.1 ± 87.9	253.2 ± 101.0	174.5 ± 87.6	180.1 ± 95.2
*p*-Value (Δ from Preoperative value)	-	0.248	0.632	**0.0034**	0.425	0.487
ADAMTS13/VWF Ratio						
	Pre-Operative	Immediate Post-Operative	POD 1	POD 2	2 weeks	6 Weeks
N	22	17	18	18	17	18
Median	0.64	0.60	0.54	0.36	0.84	0.71
Range	0.13–1.3	0.27–1.1	0.22–1.3	0.20–0.90	0.26–1.5	0.31–2.0
Mean ± SD	0.70 ± 0.32	0.65 ± 0.20	0.65 ± 0.34	0.41 ± 0.20	0.82 ± 0.39	0.88 ± 0.53
*p*-Value (Δ from Preoperative value)	-	0.338	0.120	**0.0016**	0.647	0.976

VWF, von Willebrand factor; POD, postoperative day; SD, standard deviation; N, number of patients; Δ change from baseline. Bold is statistically highly significant.

## Data Availability

The data will be available upon request.
